# Supplementary material for: Diffusion-based skin disease data augmentation with fine-grained detail preservation and interpolation for data diversity
Source: PLoS One. 2025 Oct 3;20(10):e0331404. doi: 10.1371/journal.pone.0331404 (PMC12494260; doi:10.1371/journal.pone.0331404)
Supplement: Attachment — Submitted filename: Response to Reviewers.docx [file pone.0331404.s001.docx]

**Response to Reviewers**

**Title(Revised):**
Diffusion-based skin disease data augmentation with fine-grained detail preservation and interpolation for data diversity
**Old title:** Diffusion-based skin disease data augmentation with fine-grained detail preservation and severity control

We thank the academic editor and all three reviewers for their valuable feedback and constructive comments. We have carefully revised the manuscript to address all points raised. Below, we provide a detailed point-by-point response, with reviewer comments in bold, our responses in *plain text*, and any changes to the manuscript marked in red in the revised version.
We would also like to inform the reviewers of a key change in our experimental setup, which led to slight differences in numerical results compared to the previous submission.

1. **Input resolution update.**
   In the original manuscript, input images were resized to 224 × 224. Based on the insight that larger resolutions can capture more detailed features, we revised our preprocessing to use 256 × 256 images for all experiments. This change led to slightly different absolute performance values, but the overall trends and comparative observations remain consistent**.**
2. **Improved handling of class imbalance.**
   During post-review analysis, we observed that the classifiers in our previous submission showed signs of overfitting to majority classes. This was expected, as the HAM10000 dataset is highly imbalanced (e.g., some classes have >6 000 images, others <150). To address this, we re-trained the classifiers using class**-**balanced weighting, which slightly reduced the overall accuracy but improved generalization and fairness across categories. Importantly, this correction did not alter the comparative outcomes between real and synthetic data.

**Reviewer #1**

**1. [Insufficient diversity of synthesized images: In some cases, synthesized images may overly emphasize certain details and features (such as hair areas or specific color information), resulting in images that are not natural enough and affecting the diversity and authenticity of synthesized data]**
Our response:

We agree with your observation. To address the issue of incomplete generation in hair-rich regions, we implemented the Dullrazor algorithm, a traditional hair removal method. By removing hair regions before synthesis, we confirmed that this approach can suppress excessive hair generation to some extent. This limitation and our solution are discussed in the revised manuscript (lines 623-628 in the Limitations section), with visual examples provided in Figure 11.

**2. [Insufficient diversity of synthesized images: In some cases, synthesized images may overly emphasize certain details and features (such as hair areas or specific color information), resulting in images that are not natural enough and affecting the diversity and authenticity of synthesized data]**
Our response:

We acknowledge your concern regarding the overemphasis of certain features in synthesized images. This issue is particularly pronounced when training data is limited, leading to overfitting on specific characteristics. The skin lesion domain is especially vulnerable due to the scarcity of available data compared to other domains.

To mitigate this issue, we implemented comprehensive data augmentation strategies during the training process:

- Standard augmentations: rotation and flip

- Advanced transformations: elastic transform and affine transformations

- All augmentations were applied probabilistically to maximize diversity

These augmentation techniques were implemented in the data preprocessing pipeline of the train_diffusion process, which we provide with the revision.

However, we acknowledge that despite these efforts, the absolute scarcity of training data occasionally results in inaccurate image generation. This fundamental limitation of data availability in the medical imaging domain remains a challenge.

**3. [Limitations of evaluation indicators: Although commonly used indicators such as FID and IS are used to evaluate the quality of synthesized images, these indicators show certain limitations in medical image synthesis tasks and cannot fully reflect the actual contribution of generated data to downstream diagnostic tasks.]**
Our response:
We agree with your observation regarding the limitations of FID and IS metrics in medical image synthesis. While these metrics, originally developed for GAN-based models, are commonly applied to diffusion models, recent research has questioned their suitability for non-GAN architectures. We have cited relevant studies in the revised manuscript (lines 521-528, under "Evaluation of synthetic image quality and fine detail preservation" in the Results section) that demonstrate FID and IS do not guarantee absolute correlation with downstream tasks such as classification performance. These metrics may indicate image quality but fail to capture the clinical value of synthesized data. We acknowledge that while we report FID and IS scores following conventional practice in diffusion model evaluation, these metrics are insufficient for assessing the diagnostic utility of generated medical images. Future research should focus on developing clinically-relevant metrics that quantify the basis for clinical decision-making, thereby better reflecting the actual contribution of synthetic data to diagnostic tasks.

**Reviewer #2**

**1. [The introduction does not sufficiently motivate the clinical importance of skin disease diagnosis. The authors could strengthen this section by briefly discussing the prevalence and diagnostic challenges of skin diseases, highlighting current diagnostic methods and their limitations, and incorporating relevant examples from recent literature to clearly position their synthetic data augmentation approach.]**
Our response:
Thank you for your valuable feedback. We have substantially strengthened the introduction to better motivate the clinical importance of skin disease diagnosis. The revised introduction now includes: 1. Global health burden: Added epidemiological data showing skin diseases affect 30-70% of individuals worldwide and rank as the 4th leading cause of nonfatal disease burden globally (lines 8-10). 2. Clinical significance: Emphasized the critical impact of early diagnosis, particularly for melanoma where 5-year survival rates drop from 99% with early detection to dramatically lower rates upon metastasis (lines 11-13). 3. Current diagnostic challenges: Highlighted the shortage of dermatologists in many regions and the difficulty in distinguishing between benign pigmented lesions and malignant melanoma, even for experienced specialists (lines 14-18).

**2. [The study uses a dataset of dermoscopy images but only provides class names. The authors should include brief clinical descriptions and key morphological characteristics of each class to enhance clarity and provide better context.]**
Our response: Thank you for this suggestion. We have enhanced the Datasets section by adding comprehensive clinical descriptions and morphological characteristics for each class. These additions (lines 241-279) include: - Detailed clinical descriptions of each lesion type - Key morphological features that distinguish each class - Diagnostic characteristics relevant to dermoscopic evaluation All descriptions are supported by citations from the original dataset publications to ensure accuracy and clinical relevance. This additional context will help readers better understand the complexity and diversity of the skin lesion classes used in our study.

**3. [The authors should clarify how the 5-scale CLIP embeddings are derived, as the current explanation lacks detail. While ELITE is referenced, a brief description within the relevant section would improve completeness and accessibility for readers unfamiliar with the method.]**
Our response:
Thank you for highlighting this need for clarification. We have significantly expanded the explanation of the 5-scale CLIP embeddings derivation in the "Preserving visual details through multi-level embeddings" subsection of the Materials and Methods section (lines 346-393).

The revised text now includes:

- A detailed step-by-step explanation of how the multi-scale embeddings are extracted

- The specific layers and resolutions used for each scale

- A comprehensive description of the token combination mechanism

- Mathematical formulations showing how embeddings from different layers are integrated

Additionally, we have enhanced the adapter formulation beyond simple equations to provide deeper insights into:

- The token concatenation and fusion process

- The layer-wise integration strategy

- The rationale behind our multi-scale approach

These additions ensure that readers unfamiliar with ELITE or multi-scale embedding methods can fully understand our implementation without needing to reference external sources.

**4. [The authors should provide more details on the inference process of their latent diffusion model. While Stable Diffusion is originally designed for text-to-image generation, this work leverages multi-level CLIP embeddings to preserve fine-grained details. Is this the sole conditioning mechanism, or are additional factors, such as class labels, incorporated?]**

**5. [The authors briefly mention DDIM in the related works section, however it is not mentioned again. It should be explicitly mentioned that DDIM is used during inference.]**
*Our response:*
Thank you for pointing out the need for more detailed explanation of our inference process and conditioning mechanisms. We have expanded the Implementation Details section (lines 428-464) to address both concerns:

Regarding conditioning mechanisms(#4):
We clarified that our model uses dual conditioning:
- Multi-level CLIP embeddings for fine-grained visual detail preservation
- Class label embeddings as additional conditioning to ensure class-specific generation The revised text now explicitly describes how these two conditioning signals are combined during the diffusion process.

Regarding DDIM usage (#5):
We now explicitly state that DDIM is used during inference, including:
 - The specific number of inference steps used
Additionally, we have added class-specific implementation details:
- Inference steps optimized for each lesion class
- CFG (Classifier-Free Guidance) scale values per class

**6. [Latent space manipulation assumes the generated lesion corresponds to the highest severity level, but this may not always be the case. Could the authors clarify how severity levels are defined in the latent space and whether any constraints or validation steps ensure a consistent and accurate progression of severity?]**
Our response:
We appreciate this critical observation and take it very seriously. You are correct that simple bicubic interpolation in the latent space cannot be considered a clinically valid method for representing disease severity progression. After consulting with dermatology specialists, we learned that: - Severity assessment requires comprehensive evaluation beyond what cropped lesion images can provide - Clinical severity involves multiple complex factors including not only color and size, but also border irregularity, presence of ulceration, and other morphological features - Our interpolation method cannot capture these multifaceted clinical aspects Therefore, we acknowledge that referring to our latent space manipulation as "severity control" is misleading and scientifically inaccurate. We have made the following revisions: 1. Title revision: Changed from "severity control" to "enhancing data diversity" to avoid misrepresentation 2. Content revision: Removed all claims about severity control throughout the manuscript 3. Repositioning: Clarified that latent space interpolation serves solely as a data augmentation technique for increasing sample diversity, not as a clinical severity modeling tool We have concluded that the interpolation-based method should be considered primarily as a means to expand data diversity rather than for severity control. This approach is more scientifically sound and better reflects the actual capabilities and appropriate use cases of our method.

**7. [The 8-channel VAE is evaluated using LPIPS and MS-SSIM for reconstruction, but its impact on the latent space is not assessed. Increasing VAE channels enlarges the latent representation and UNet, potentially improving performance but at a higher computational cost. An alternative is increasing input resolution while maintaining the same downsampling ratio, expanding the latent space without modifying the architecture. An ablation study comparing these approaches would be necessary to better justify the choice of an 8-channel VAE.]**
Our response: Thank you for this insightful suggestion regarding the comparison between channel expansion and spatial resolution increase. We appreciate your comprehensive analysis of the trade-offs involved. We initially planned experiments following your suggested approach, testing resolutions from 224×224 to 256×256 and up to 512×512. However, we encountered significant computational constraints: even with 384×384 input and batch size of 1, training time increased by over 30% and nearly exhausted our available resources. Given our laboratory's computational limitations, 256×256 proved to be the practical upper limit for systematic experimentation. We agree that higher resolutions could better capture fine-grained details, as you noted. This remains an important direction for future work when additional computational resources become available. We plan to conduct comprehensive ablation studies comparing: - Higher spatial resolutions (384×384, 512×512) with standard 4-channel VAE - Our current 8-channel VAE approach at various resolutions Regarding the VAE evaluation metrics, we focused on LPIPS and MS-SSIM because, in our current framework, the VAE functions primarily as an input-output reconstruction module. These metrics directly assess the visual and structural similarity essential for this role. We acknowledge that a more comprehensive evaluation of the latent space properties would provide additional insights, particularly regarding the trade-offs between channel expansion and spatial resolution. This will be addressed in our future comparative studies.

**8. [The proposed LDM is compared against Stable Diffusion as a baseline, but it is unclear how it was adapted to align with the study's objectives. The authors should provide more details on any modifications made, including changes to conditioning mechanisms and training procedures to ensure a fair and meaningful comparison. ]**
Our response:
Thank you for highlighting the need for clarity regarding our baseline comparison. We have expanded the Implementation Details section to provide comprehensive information about how Stable Diffusion was adapted for our study. For the baseline Stable Diffusion model, we:
1. Maintained standard architecture: Used the original Stable Diffusion architecture with recommended parameters to ensure reproducibility
2. Modified conditioning mechanism: Replaced text embeddings with class label embeddings to align with our medical image generation task
3. Kept training procedures consistent: Used identical training hyperparameters, data augmentation strategies, and optimization settings for both baseline and proposed models The key difference between our approach and the baseline lies in:
- Baseline: Single-scale conditioning using only class label embeddings
- Our method: Multi-scale CLIP embeddings combined with class label embeddings for enhanced detail preservation Detailed implementation specifications, including learning rates, batch sizes, and training schedules, have been added to the Implementation Details section (lines 428-464).

**9. [ The classification performance evaluation focuses on relatively shallow networks. Is there a reason deeper or transformer-based models were not considered? Given their improved feature extraction capabilities, could they bridge the 3% gap even without synthetic data augmentation? A complete justification and explanation is necessary]**
Our response:
Thank you for this valuable suggestion. Following your recommendation, we conducted additional experiments with transformer-based models and deeper CNN architectures. (line 554). We have added these results to the revised manuscript. Our initial choice of relatively shallow networks was deliberate, based on the following considerations:
- Dataset characteristics : HAM10000 has limited data quantity, with some classes being severely underrepresented. This data scarcity particularly affects minority classes, making it challenging for parameter-heavy models to generalize effectively.

- The primary value of synthetic data augmentation lies not in improving performance on well-represented classes, but in addressing the critical shortage of rare disease samples. Our approach specifically targets this class imbalance problem.

**Minor Issues:
● \mathcal{} should be used to properly format variables.
● Starting sentences with “And” should be avoided for better readability.
● LPIPS and MS-SSIM are both missing citations.**

**Reviewer #3**

**1. [The model’s performance is validated exclusively on the HAM10000 dataset, which may not capture the full variability of skin conditions across populations and imaging conditions. If possible, broader validation on diverse datasets may help to establish generalizability.]**
Our response:
We appreciate your concern regarding the generalizability of our approach. You raise an important point about validation across diverse datasets. Currently, publicly available dermoscopy datasets for skin diseases are limited, with ISIC, HAM10000, and PH2 being the most prominent.
However, these datasets present significant challenges:
- Limited data quantity or restricted class diversity
- Substantial overlap between datasets due to shared data collection sources (hospitals, institutions)
- Insufficient representation of diverse populations and imaging conditions This overlap makes cross-dataset validation less meaningful, as it would not truly test generalization to new domains but rather to redundant samples from similar sources. To address this fundamental limitation, we are actively collecting our own diverse dataset with:
- Broader population representation
- Varied imaging conditions and devices
- Severity-based annotations for more nuanced classification
This new dataset is currently under development and will be utilized in our future research to properly validate the generalizability of synthetic data augmentation approaches across truly independent data sources.

**2. [The study lacks comprehensive ablation experiments to evaluate the individual impact of architectural choices (e.g., multi-level embeddings, adapter layers). This omission limits the interpretability and reproducibility of the work.]**
Our response:
Thank you for highlighting the need for comprehensive ablation studies. We have added a new section Ablation on Channel Width and Multi-level Embeddings (lines 581-599) that systematically evaluates the individual contributions of our key architectural components. The ablation study includes: - Diffusion generation results with varying VAE channels (4-channel vs. 8-channel) - Generation results with and without multi-level embeddings We acknowledge certain architectural constraints in our ablation design: 1. VAE and Diffusion pipeline: As our model is based on LDM architecture, the VAE module and diffusion pipeline are inherently coupled and cannot be independently removed. Therefore, we focused on comparing different VAE channel configurations rather than removing the VAE entirely. 2. Adapter layers: These layers are integral to injecting multi-level embeddings into the diffusion U-Net and cannot be meaningfully separated from the multi-level embedding system. The adapter architecture is specifically designed for this injection mechanism.

**3. [The background section would benefit from the addition of other relevant medical literature, such as "Advancing Precision Medicine: VAE Enhanced Predictions of Pancreatic Cancer Patient Survival in Local Hospital" , to strengthen its persuasiveness.]**
Our response:

Thank you for suggesting this highly relevant reference. We have incorporated "Advancing Precision Medicine: VAE Enhanced Predictions of Pancreatic Cancer Patient Survival in Local Hospital" into the Related Work/Medical data augmentation section (lines 138-142).
This paper is particularly pertinent to our work as it:
- Demonstrates VAE application for augmenting limited medical data - Addresses data scarcity challenges in pancreatic cancer patient datasets
- Validates the effectiveness of VAE-based augmentation in medical contexts.
The inclusion of this reference strengthens our literature review by providing additional evidence for VAE's utility in medical data augmentation, particularly for conditions with limited patient data. This aligns perfectly with our approach of using diffusion models for skin lesion data augmentation, as both works address the fundamental challenge of data scarcity in medical imaging and diagnostics.
