# Supplementary material for: Diffusion-based skin disease data augmentation with fine-grained detail preservation and interpolation for data diversity
Source: PLoS One. 2025 Oct 3;20(10):e0331404. doi: 10.1371/journal.pone.0331404 (PMC12494260; doi:10.1371/journal.pone.0331404)
Supplement: Attachment — Submitted filename: Response_to_Reviewers_auresp_2.docx [file pone.0331404.s002.docx]

**Response to Reviewers**

**Title:**
Diffusion-based skin disease data augmentation with fine-grained detail preservation and interpolation for data diversity

We thank the academic editor and all three reviewers for their valuable feedback and constructive comments. We have carefully revised the manuscript to address all points raised. Below, we provide a detailed point-by-point response, with reviewer comments in bold, our responses in plain *text*, and any changes to the manuscript marked in red in the revised version.
==========================================================
We would also like to inform the reviewers of key changes and additions in our revised manuscript:

1. **Terminology refinement.**

Following reviewers' suggestions, we have removed all instances of "proxy severity level" and replaced them with more accurate terms such as "latent space interpolation between normal and lesion appearances" to clarify that our method is intended solely for visual data augmentation, not clinical severity assessment.

2. **Extended experimental validation.**

We have significantly expanded our experiments to include: (a) broader architecture evaluation with additional modern models (EVA and CoAtNet, beyond the previously tested ConvNeXt and Swin Transformer), (b) cross-dataset zero-shot evaluation on PAD-UFES-20 to demonstrate generalization beyond dermoscopic images, and (c) quantitative diversity analysis using OpenCLIP embeddings to validate the utility of interpolated samples.

3. **Additional ablation studies**

We supplemented our ablation analysis with a new examination of the class-conditioning strategy, including experiments across various CFG scales, showing how class-conditioning reflects the distinctive characteristics of each individual class.
==========================================================

Detailed responses to each reviewer's specific comments are provided on the following pages.

**Reviewer #2**

**1. “A minor remaining concern is the use of the term 'proxy severity level.' While an improvement over 'severity control,' the term 'proxy' implies an indirect measure that has been validated to correlate with the ground truth. As the authors rightly acknowledge, the proposed latent space interpolation method does not capture the multifaceted nature of clinical severity and has not been validated as such. I recommend replacing all instances of 'proxy severity level' with a more accurate and defensible term, such as the one used in the rebuttal: 'latent space interpolation for enhanced data diversity”**
Our response:
We thank the reviewer for pointing this out.

In response, we have carefully revised the manuscript to remove all instances of the term “proxy severity level” and related phrases.

Wherever applicable, we have replaced them with more accurate and defensible expressions such as “latent space interpolation between normal and lesion appearances” or “smoothly varying visual characteristics”, as suggested by the reviewer through the example phrase “latent space interpolation for enhanced data diversity.”
These revisions were made to clarify that the interpolation method is intended solely for visual data augmentation and is not meant to imply any clinical interpretation regarding disease severity. The manuscript has also been revised to avoid terminology that could be interpreted as suggesting alignment with clinically defined severity scales.

**Reviewer #5**

**1. [First, the notion of “proxy severity” lacks sufficient empirical support. While the authors acknowledge that the interpolation does not represent clinically validated severity levels, no evaluation—either quantitative or by domain experts—is provided to demonstrate the interpretability or utility of these interpolated images.]**
Our response:
We thank the reviewer for this important comment. We acknowledge that the expression “proxy severity level” could be misinterpreted as implying alignment with clinically validated severity scales. To avoid such misinterpretation, we have removed all instances of the term “proxy severity level” and related phrases from the manuscript. Wherever applicable, we replaced them with more precise and defensible expressions such as “latent space interpolation between normal and lesion appearances” or “smoothly varying visual characteristics,” clarifying that our approach is intended solely for visual data augmentation rather than clinical severity modeling.

In addition, to address the reviewer’s concern regarding empirical support, we have incorporated a new quantitative analysis to examine the potential utility of interpolated images from a data diversity perspective. Specifically, we compared two synthetic image sets—A (500 randomly generated samples) and A_I_ (the same set with half replaced by interpolated samples)—by extracting CLIP embeddings, projecting them into two dimensions using PCA, and calculating semantic variance for each of the seven classes (Table.6). Across all classes, A_I_ exhibited higher variance than A, indicating that interpolation-based samples captured a broader range of semantic characteristics.

The results in Tableㅋ` 6 show that variance increases ranged from +0.45 (Class 1) to +2.86 (Class 4), with the largest gains observed in classes that are relatively underrepresented in the original dataset. This pattern suggests that latent space interpolation may be particularly effective in enhancing diversity for minority classes, thereby improving the coverage of the feature space available to the model during training.

The corresponding visualizations (Fig. 12) further illustrate that, in most classes, A_I_ samples (triangles) are more widely dispersed in the embedding space than A samples (circles), suggesting an expansion of the representational space that may contribute to enhanced training data diversity.
These results provide quantitative indications that the interpolation approach can contribute to visual data augmentation.

**2. [Second, the study is limited to a single dataset (HAM10000), which restricts its generalizability. Although the authors discuss the limitations of existing public datasets, even a small-scale test on a secondary dataset (e.g., PH2 or ISIC) would help establish broader relevance.]**
Our response:
We appreciate the reviewer’s valuable suggestion regarding the need for broader validation beyond the HAM10000 dataset. In the revised manuscript, we have incorporated an additional **cross-dataset zero-shot evaluation** using the publicly available PAD-UFES-20 dataset to further examine the generalization capability of our classifiers.

While other dermoscopic datasets such as PH2 and ISIC are publicly available, they share common data collection sources with HAM10000, resulting in substantial image overlap. This overlap makes them less suitable as independent test sets for evaluating cross-dataset performance, as duplicated samples could confound the interpretation.

Therefore, following the reviewer’s suggestion, we opted for the PAD-UFES-20 dataset, which—while still within the dermatology domain—contains clinical photographs captured using smartphone cameras rather than professional dermoscopic images (Fig. 8). This setup enables evaluation under more diverse imaging conditions, including variations in acquisition devices, lighting, resolution, and background context.

For a fair comparison, we evaluated only the four classes common to both datasets: AKIEC, BCC, NV, and MEL. Nine classification models trained solely on HAM10000 (without fine-tuning) were directly tested on PAD-UFES-20. As shown in Table. 4 transformer-based architectures such as EVA(Acc=64.27%) and Swin Transformer (Acc=62.15%) showed relatively stronger robustness to domain shifts compared to conventional CNN-based models. While some performance differences were observed compared to the in-domain HAM10000 results, the relative ranking of models remained consistent, suggesting that our approach maintains its effectiveness under varied imaging conditions.

**3. [Additionally, while multiple classifier backbones are tested, the evaluation primarily focuses on shallow models. It remains unclear whether performance improvements would persist with more powerful architectures that could compensate for limited data without augmentation.]**
Our response:

We thank the reviewer for this valuable comment. In the revised manuscript, we have expanded our downstream classification experiments to include a broader spectrum of architectures, ranging from relatively simple CNN models (VGG13, ResNet18) to deeper and more modern architectures such as ConvNeXt, Swin Transformer, EVA, and CoAtNet. The updated results are provided in Table.3 (Section “Classification downstream task”).

Our results show that, across both simpler and more modern backbone models, adding synthetic data generally improves classification accuracy. While the magnitude of improvement varies by architecture, this suggests that the proposed augmentation approach can contribute to enhancing performance regardless of model complexity. Notably, modern architectures such as Swin Transformer, ConvNeXt, EVA, and CoAtNet achieved higher absolute performance than earlier-generation models.

however, in the case of EVA, the improvement from adding synthetic data was relatively modest (less than 3%). This suggests that certain models may already extract sufficiently rich representations from the original data, leaving relatively less room for further gains through synthetic augmentation alone. These findings suggest that our method is effective not only for shallow networks but also for more complex architectures, although the degree of benefit from synthetic augmentation can vary depending on the model.

**4. [The ablation study adds value but does not fully isolate the contributions of each component, such as adapter layers or class-conditioning strategies.]**
Our response:

We appreciate the reviewer’s comment on the need to more clearly isolate the contributions of individual components.

However, our design intentionally integrates the adapter and the multi-level embedding pathway to function as a unified mechanism. The adapter layers are crucial for injecting multi-level embeddings into the diffusion U-Net by performing cross-attention. As a result, removing the adapter would inherently disable this pathway, making it technically difficult to evaluate the two components independently.

Therefore, in our ablation study, the comparison between the 8-channel VAE without both the multi-level embeddings and adapter layers (“8ch”) and the 8-channel VAE with both components included (“8ch+ML”) captures their combined impact.

In addition, we appreciate and agree with the reviewer's comment about class-conditioning strategies. To address this valid point, we have supplemented our ablation study with an additional analysis of the class-conditioning strategy.

Specifically, we generated samples for all lesion classes using identical random seeds and compared results across various CFG (Classifier-Free Guidance) scales, including a non-conditioned setting in which the class label embedding was entirely removed during sampling. As shown in the newly added Figure 10, removing class-conditioning weakened class-specific lesion patterns and led to more ambiguous class characteristics. In contrast, increasing the CFG value emphasized distinctive morphological features for each class (e.g., pigment networks in melanoma and vascular lacunae in vascular lesions). These results qualitatively confirm that class-conditioning contributes to maintaining class-specific characteristics.

**5. [Furthermore, the choice to expand VAE channels rather than image resolution is justified based on computational constraints, yet no comparative results are shown. More concrete evidence on the trade-offs between spatial resolution and latent dimensionality would be useful.]**
Our response:

We appreciate the reviewer’s comment on the need to clarify the trade-offs between expanding VAE channels and increasing image resolution. Although we could not conduct a full empirical comparison due to hardware limitations, we provide here a mathematical estimation of the memory requirements and computational complexity for two representative configurations:

| Configuration | Input Resolution | VAE Channels | Latent Map Size | Latent Elements | Latent Memory | Spatial  Tokens |
| --- | --- | --- | --- | --- | --- | --- |
| 256^2^, 8ch | 256×256 | 8 | 32×32×8 | 8,192 | 1 x | 1 x |
| 512^2^, 4ch | 512×512 | 4 | 64×64×4 | 16,384 | 2 x | 4 x |

From this estimation, the 512×512, 4-channel setup doubles the number of latent elements (and thus latent-storage memory) relative to the 256×256, 8-channel setup. More importantly, the spatial token count quadruples, so convolutional layers scale by roughly ~4×, cross-attention by ~4**×** (with fixed text length), and global self-attention by up to ~16**×** in FLOPs. In practice, when intermediate activations and attention buffers are included, we typically observe ~2.5–3**×** higher VRAM usage, which exceeded our available GPU memory even with a batch size of one. Given these constraints, we chose the 256×256, 8-channel configuration to increase latent representational capacity while remaining computationally feasible and maintaining stable training.

We acknowledge that a direct empirical comparison would further strengthen the justification. In future work, we plan to revisit this by upgrading hardware, applying memory-efficient attention, gradient checkpointing, and mixed precision, and benchmarking 4-channel VAEs against 8-channel latent-width models across reconstruction quality, generative fidelity, and downstream performance on high-resolution images.

**6. [Evaluation metrics are another concern. The continued reliance on FID and IS—despite acknowledging their limitations in medical imaging—underscores the need for more appropriate alternatives. Even a basic human visual assessment or task-based evaluation (e.g., classification accuracy using only synthetic images) would strengthen the argument that these images are diagnostically useful.]**
Our response:

We agree with the reviewer’s observation regarding the limitations of FID and IS in evaluating the diagnostic utility of medical images. While these metrics, originally developed for GAN-based models, are still conventionally applied to diffusion models for comparability, they primarily reflect distributional similarity and do not fully capture the clinical value of synthesized data.

To complement these limitations, our evaluation includes classification performance in real-only, synthetic-only, and mixed settings, which can serve as task-based evidence regarding the utility of the generated images. These results indicate that while synthetic-only training performs below real-only, combining synthetic and real images generally improves accuracy, suggesting that the generated data may be useful as complementary training data rather than as a full substitute for real data.

In this revision, we additionally included a cross-dataset zero-shot evaluation on the PAD-UFES-20 dataset (Table 4, Fig. 8) to examine generalization under different image acquisition settings (professional dermoscopic devices vs. smartphone cameras), as well as an OpenCLIP-based analysis (PCA visualizations and per-class semantic variance; Fig. 12, Table 6) comparing interpolated and randomly generated samples. While FID and IS remain the most commonly reported metrics for image generation, these supplementary evaluations can provide complementary quantitative perspectives that may help address their known limitations, particularly in the context of medical image synthesis.

In parallel with this work, we are building an expanded skin lesion dataset with dermatologist-validated diagnostic and severity annotations. In future research, we aim to leverage this dataset to conduct blinded expert review protocols (e.g., lesion realism, artifact assessment, diagnostic plausibility) to more precisely quantify the clinical utility of the generated images.
